# A Novel Long Non-coding RNA, MSTRG.51053.2 Regulates Cisplatin Resistance by Sponging the miR-432-5p in Non-small Cell Lung Cancer Cells

**DOI:** 10.3389/fonc.2020.00215

**Published:** 2020-02-25

**Authors:** Jie Zhang, Chuanqin Xu, Yan Gao, Yi Wang, Zongli Ding, Yueming Zhang, Wenyi Shen, Yulong Zheng, Yufeng Wan

**Affiliations:** ^1^Department of Respiratory Diseases, The Affiliated Huai'an Hospital of Xuzhou Medical University, Huai'an, China; ^2^Department of Respiratory Diseases, The Lianshui County People's Hospital, Huai'an, China

**Keywords:** non-small cell lung cancer, cisplatin resistance, lncRNA, GSH, transcriptome, competing endogenous RNA

## Abstract

**Objective:** The aim of this study was to investigate the molecular mechanisms underlying cisplatin (DDP) resistance in non-small cell lung cancer (NSCLC) cells by constructing a competing endogenous RNA (ceRNA) network.

**Methods:** The gene expression profiles of human lung adenocarcinoma DDP-resistant cell line (A549/DDP) and its progenitor (A549) were comparatively evaluated by whole-transcriptome sequencing. The differentially expressed genes (DEGs) were subjected to KEGG pathway analysis. The expression levels of mRNAs involved in several pathways associated with conferring DDP resistance to tumor cells were evaluated. The ceRNA network was constructed based on the mRNA expression levels and the sequencing data of miRNA and lncRNA. Several ceRNA regulatory relationships were validated.

**Results:** We quantified the expression of 17 genes involved in the six pathways by quantitative real-time polymerase chain reaction (qRT-PCR). The differential protein expression levels of eight genes were quantified by western blotting. Western blot analysis revealed six differentially expressed proteins (MGST1, MGST3, ABCG2, FXYD2, ALDH3A1, and GST-ω1). Among the genes encoding these six proteins, *ABCG2, ALDH3A1, MGST3*, and *FXYD2* exhibited interaction with 8 lncRNAs and 4 miRNAs in the ceRNA regulatory network. The expression levels of these lncRNAs and miRNAs were quantified in cells; among these, 6 lncRNAs and 4 miRNAs exhibited differential expression between A549/DDP and A549 groups, which were used to construct a ceRNA network. The ceRNA regulatory network of MSTRG51053.2-miR-432-5p-*MGST3* was validated by luciferase reporter assay.

**Conclusion:** The MSTRG51053.2 lncRNA may function as a ceRNA for miR-432-5p to regulate the DDP resistance in NSCLC. The *MGST1, MGST3, GST-*ω*1*, and *ABCG2* mRNAs, miR-432-5p and miR-665 miRNAs, and MSTRG51053.2 and PPAN lncRNAs can serve as potential DDP drug targets to reverse DDP resistance in NSCLC.

## Introduction

Globally, lung cancer is the leading cause of cancer-related deaths (1). Non-small cell lung cancer (NSCLC) accounts for about 85% of all lung cancer cases (2). Most of the patients with NSCLC are diagnosed at advanced stage and exhibit a 5-year survival rate of only 17.4% (1, 2). Currently, the first line of treatment for patients with NSCLC, especially advanced stage cases, is cisplatin (DDP)-based chemotherapy (3, 4). DDP exhibits cytotoxicity by interacting with the DNA to form platinum-DNA adducts, which inhibit DNA replication (5, 6). However, tumors exhibit resistance to DDP chemotherapy, which is a major impediment to successful chemotherapy. The mechanism underlying tumor resistance to DDP is complex. Hence, there is an urgent need to elucidate this mechanism.

The proposed mechanisms underlying the acquired resistance of tumor to DDP mainly include cytoplasmic sequestration, decreased cytoplasmic accumulation, and enhanced DNA repair (7, 8). Several genetic factors are reported to contribute to the drug resistance of tumors. Mutations in genes, such as Kirsten rat sarcoma viral oncogene (KRAS) (9), tumor suppressor protein 53 (TP53) (10), and PI3-kinase subunit alpha (PIK3CA) (11) are associated with conferring drug resistance to lung cancer cells. Additionally, non-coding RNAs, such as microRNA (miRNA) and long non-coding RNA (lncRNA) are reported to mediate drug resistance in lung cancer patients undergoing chemotherapy treatment (12, 13). The critical role of various RNAs, including mRNAs, miRNAs, and lncRNAs in conferring DDP resistance to NSCLC cells was elucidated with the development of transcriptome sequencing and microarray technologies. Recent studies have demonstrated that lncRNAs can function as competing endogenous RNAs (ceRNAs) to regulate the downstream functions by sponging the target miRNAs (14, 15). To the best of our knowledge, there are no studies that have evaluated the role of ceRNAs in conferring DDP resistance to NSCLC cells.

In this study, we compared the gene expression profile between a human lung adenocarcinoma DDP-resistant cell line (A549/DDP) and its progenitor (A549 cells) using whole-transcriptome sequencing. Additionally, we selected several pathways associated with conferring DDP resistance to NSCLC cells and examined the mRNA expression levels of genes involved in these pathways. Based on the mRNA expression levels and the sequencing data of miRNA and lncRNA, the ceRNA network was constructed and validated several ceRNA regulatory relationships. Our results may help to elucidate the potential molecular mechanism underlying DDP resistance in NSCLC cells.

## Results

### Differential Expression Analysis and KEGG Pathway Analysis of mRNAs

**Figure 1:**
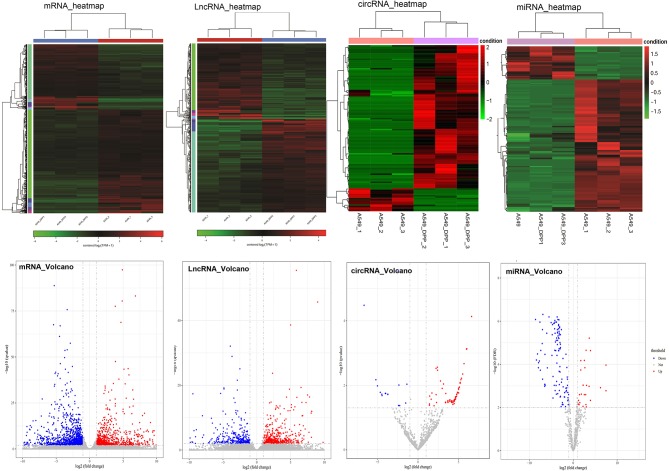
The heatmaps and volcano pictures of these differentially expressed mRNAs, lncRNAs, circRNA, and miRNAs. Each cell line was repeated for three times (A549_1/2/3 and A549/DPP_1/2/3).

After analysis, a total of 882 downregulated and 554 upregulated mRNAs, 219 downregulated and 273 upregulated lncRNAs, 106 downregulated, and 17 upregulated circRNAs, as well as 89 downregulated and 15 upregulated miRNAs were identified between A549/DDP and A549 groups. The heatmaps and volcano pictures of these differentially expressed genes are shown in Figure 1.

KEGG pathway analysis of the differentially expressed mRNAs revealed 27 significantly upregulated pathways and 54 significantly downregulated pathways (Table S1). We focused on 6 pathways that are associated with conferring DDP resistance to tumor cells, including bile secretion (ko04976), metabolism of xenobiotics by cytochrome P450 (ko00980), glutathione metabolism (ko00480), drug metabolism-cytochrome P450 (ko00982), platinum drug resistance (ko01524), and chemical carcinogenesis (ko05204). As shown in Table 1, there were 17 genes involved in these 6 pathways.

**Table 1:** The 17 genes involved in the six pathways associated with the resistance to cisplatin.

**GeneID**	**Gene name**	**log2fold change**	**q value**	**Gene description**
ENSG00000113161	HMGCR	2.78	3.31E-05	3-hydroxy-3-methylglutaryl-CoA reductase
ENSG00000118777	ABCG2	1.65	2.45E-08	ATP binding cassette subfamily G member 2
ENSG00000186198	*SLC51B*	4.67	3.63E-05	Solute carrier family 51 beta subunit
ENSG00000073060	*SCARB1*	4.14	7.76E-11	Scavenger receptor class B member 1
ENSG00000137731	FXYD2	3.93	1.79E-16	FXYD domain containing ion transport regulator 2
ENSG00000136881	*BAAT*	3.20	4.77E-11	Bile acid-CoA:amino acid N-acyltransferase
ENSG00000162104	*ADCY9*	1.54	2.62E-10	Adenylate cyclase 9
ENSG00000104267	*CA2*	4.21	3.70E-06	Carbonic anhydrase 2
ENSG00000008394	MGST1	1.84	0.005926478	Microsomal glutathione S-transferase 1
ENSG00000143198	MGST3	2.86	0.00076915	Microsomal glutathione S-transferase 3
ENSG00000108602	ALDH3A1	2.44	0.000218806	Aldehyde dehydrogenase 3 family member A1
ENSG00000148834	GST-ω	1.05	0.004982697	Glutathione S-transferase omega 1
ENSG00000084207	GST-π	1.77	0.000870743	Glutathione S-transferase pi 1
ENSG00000159231	CBR3	2.09	1.22E-05	Carbonyl reductase 3
ENSG00000244122	*UGT1A7*	2.80	1.12E-08	UDP glucuronosyltransferase family 1 member A7
ENSG00000187134	*AKR1C1*	3.04	1.89E-09	Aldo-keto reductase family 1 member C1
ENSG00000100031	GGT1	1.39	0.00451235	Gamma-glutamyltransferase 1

### Expression Levels of 17 Genes in Cell Lines and Xenografted Tumor Tissues

**Figure 2:**
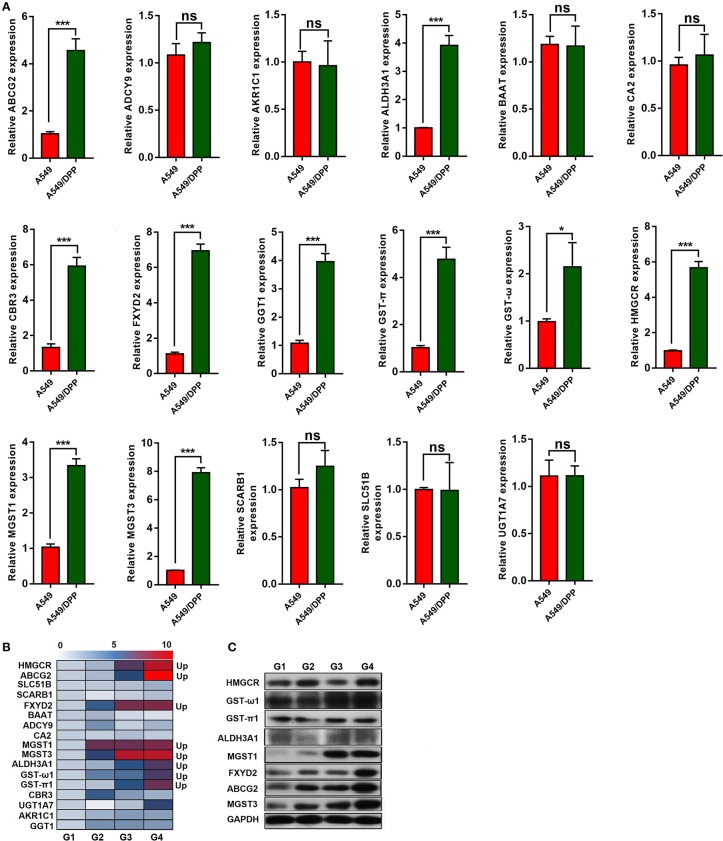
**(A,B)** The expression levels of 17 messenger RNAs (mRNAs) in the A549/DDP and A549 cells and G1-G4 tumor tissues were detected by quantitative real-time polymerase chain reaction (qRT-PCR). **(C)** The protein expression levels of 8 mRNAs in the G1-G4 tumor tissues. *P < 0.05, ***P < 0.001.

The expression levels of 17 genes in the cells and transplanted tumor tissues from the mice were quantified using quantitative real-time polymerase chain reaction (qRT-PCR). As shown in Figure 2A, the expression levels of ABCG2, ALDH3A1, CBR3, FXYD2, GGT1, GST-π, GST-ω, HMGCR, MGST1, and MGST3 were significantly upregulated in the A549/DDP group when compared to those in the A549 group. Additionally, eight genes (ABCG2, ALDH3A1, FXYD2, GST-π1, GST-ω1, HMGCR, MGST1, and MGST3) were upregulated among the G1-G4 DDP-resistant tumor tissues (Figure 2B).

The protein expression levels of these eight genes were quantified by western blotting. As shown in Figure 2C, the protein expression levels of MGST1, MGST3, ABCG2, and FXYD2 differed among the G1-G4 DDP-resistant tumor tissues. Additionally, the protein expression levels of ALDH3A1 and GST-ω1 varied among the G1-G4 DDP-resistant tumor tissues.

### ceRNA Network Construction

**Figure 3:**
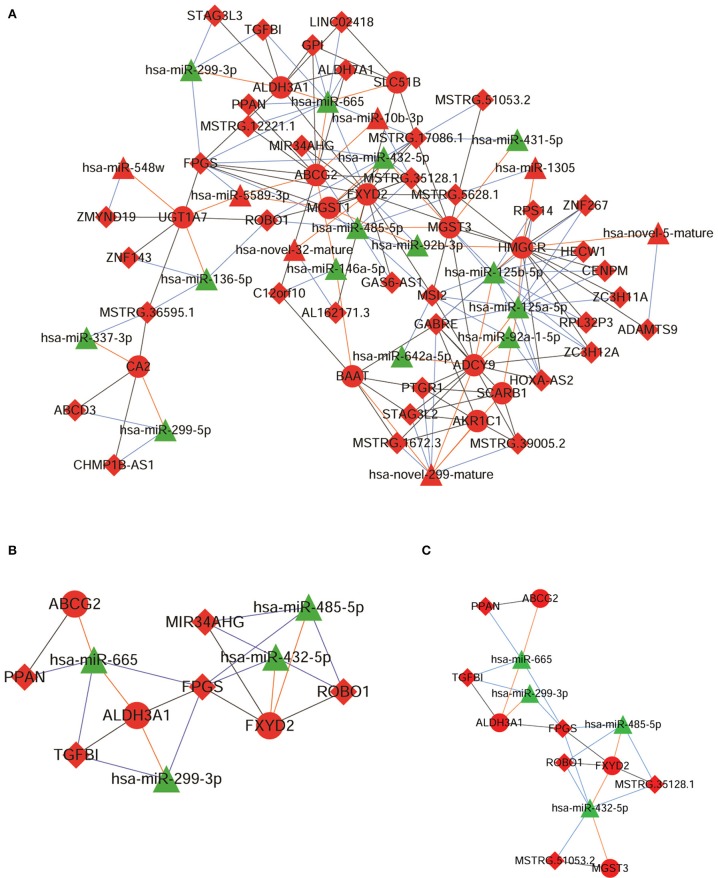
The competing endogenous RNA (ceRNA) networks constructed using **(A)** 13 messenger RNAs (mRNAs), **(B)** 4 mRNAs, 5 long non-coding RNAs (lncRNAs), and 4 micro RNAs (miRNAs), and **(C)** 6 lncRNAs and 4 miRNAs. Red nodes represent up-regulated expression, green nodes represent down-regulated expression, triangular nodes represent miRNA, circular nodes represent mRNA, and diamond nodes represent lncRNA.

A ceRNA network was constructed based on the 17 genes involved in the six pathways. Among the 17 genes, only 13 genes exhibited regulatory relationship with the differentially expressed miRNAs. The constructed ceRNA network is shown in Figure 3A.

From this ceRNA network, we extracted the ceRNA networks associated with the 6 proteins that were differentially expressed among the G1-G4 DDP-resistant xenografted tumor tissues. Among the genes encoding these six proteins, we observed that 4 genes (ABCG2, ALDH3A1, MGST3, and FXYD2) were involved in the ceRNA regulatory network comprising 8 lncRNAs (PPAN, FPGS, ROBO1, MIR34AHG, TGFBI, MSTRG.12221.1, MSTRG.35128.1, and MSTRG.51053.2) and 4 miRNAs (miR-665, miR-432-5p, miR-485-5p, and miR-299-3p) (Table S2). Finally, a ceRNA subnetwork comprising 4 mRNAs, 5 lncRNAs, and 4 miRNAs was obtained, which is shown in Figure 3B.

### Expression Levels of 8 lncRNAs and 4 miRNAs in Cell Lines and ceRNA Network Construction

**Figure 4:**
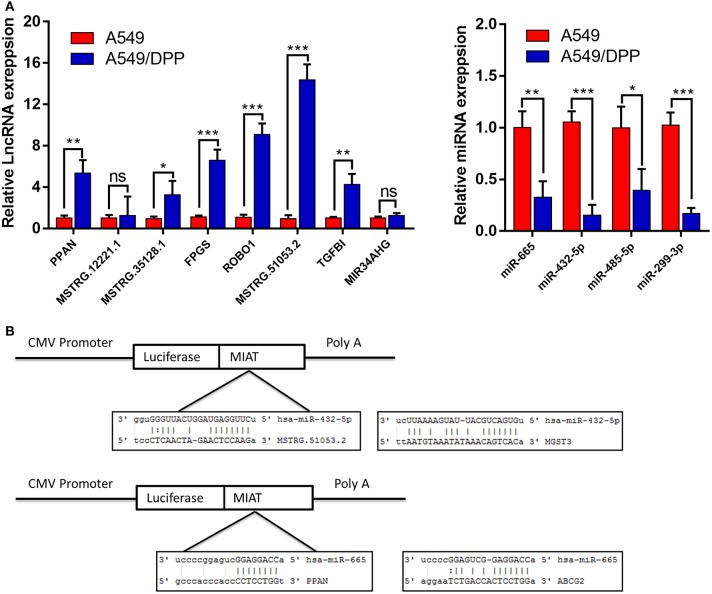
**(A)** The expression levels of 8 long non-coding RNAs (lncRNAs) and 4 micro RNAs (miRNAs) involved in the ceRNA network of the A549/DDP and A549 cells were detected by quantitative real-time polymerase chain reaction (qRT-PCR). **(B)** The binding sites involved in the competing endogenous RNA (ceRNA) regulatory networks of MSTRG51053.2-miR-432-5p-*MGST3* and PPAN-miR-665-*ABCG2*. *P < 0.05, **P < 0.01, ***P < 0.001.

The expression levels of 8 lncRNAs and 4 miRNAs in the A549 and A549/DDP cells were quantified by qRT-PCR. As shown in Figure 4A, except for MSTRG.12221.1 and MIR34AHG, the expression levels of 6 lncRNAs and 4 miRNAs were significantly different between the A549/DDP and A549 groups. A ceRNA network was constructed comprising 6 lncRNAs and 4 miRNAs, which is shown in Figure 3C. This network included 12 ceRNA networks (Table S3). Among the 12 ceRNA networks, we selected the MSTRG.51053.2-miR-432-5p-MGST3 and PPAN- miR-665-ABCG2 regulatory networks for further analysis as there is a correlation between DDP resistance and MGST and ABCG families. The binding sites involved in the two ceRNA regulatory networks are shown in Figure 4B.

### Expression Levels of Genes Involved in the MSTRG.51053.2-miR-432-5p-MGST3 and PPAN-miR-665-ABCG2 ceRNA Regulatory Networks

**Figure 5:**
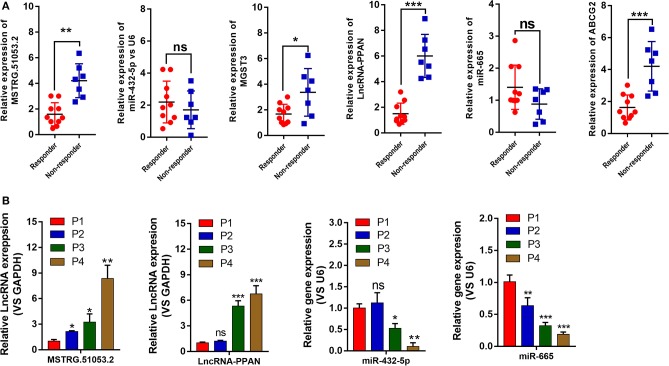
**(A)** The expression levels of MSTRG510532, PPAN, *MGST3, ABCG2* miR-432-5p, and miR-665 in the responder and non-responder groups from patients were detected by quantitative real-time polymerase chain reaction (qRT-PCR). **(B)** The expression levels of MSTRG510532, PPAN, miR-432-5p, and miR-665 in the G1-G4 tumor tissues from mice. *P < 0.05, **P < 0.01, ***P < 0.001.

As shown in Figure 5A, the expression levels of MSTRG.51053.2, PPAN, MGST3, and ABCG2 in the non-responder group were significantly higher than those in the responder group. The expression levels of miR-432-5p and miR-665 in the non-responder group were lower than those in the responder group but the difference was not statistically significant. Furthermore, the expression levels of 6 genes in the G1-G4 xenografted tumor tissues were also quantified by qRT-PCR. As shown in Figure 5B, the expression level of MSTRG.51053.2 was significantly upregulated from P2 to P4, while that of PPAN was significantly upregulated from P3 to P4. The expression level of miR-432-5p was significantly downregulated from P3 to P4, while that of miR-665 was significantly downregulated from P2 to P4. The expression levels of MGST3 and ABCG2 in the G1-G4 tumor tissues are shown in Figure 2B.

### Effects of MSTRG.51053.2 Overexpression on the Expression of miR-432-5p and MGST3 and on DDP Sensitivity of A549 Cells

**Figure 6:**
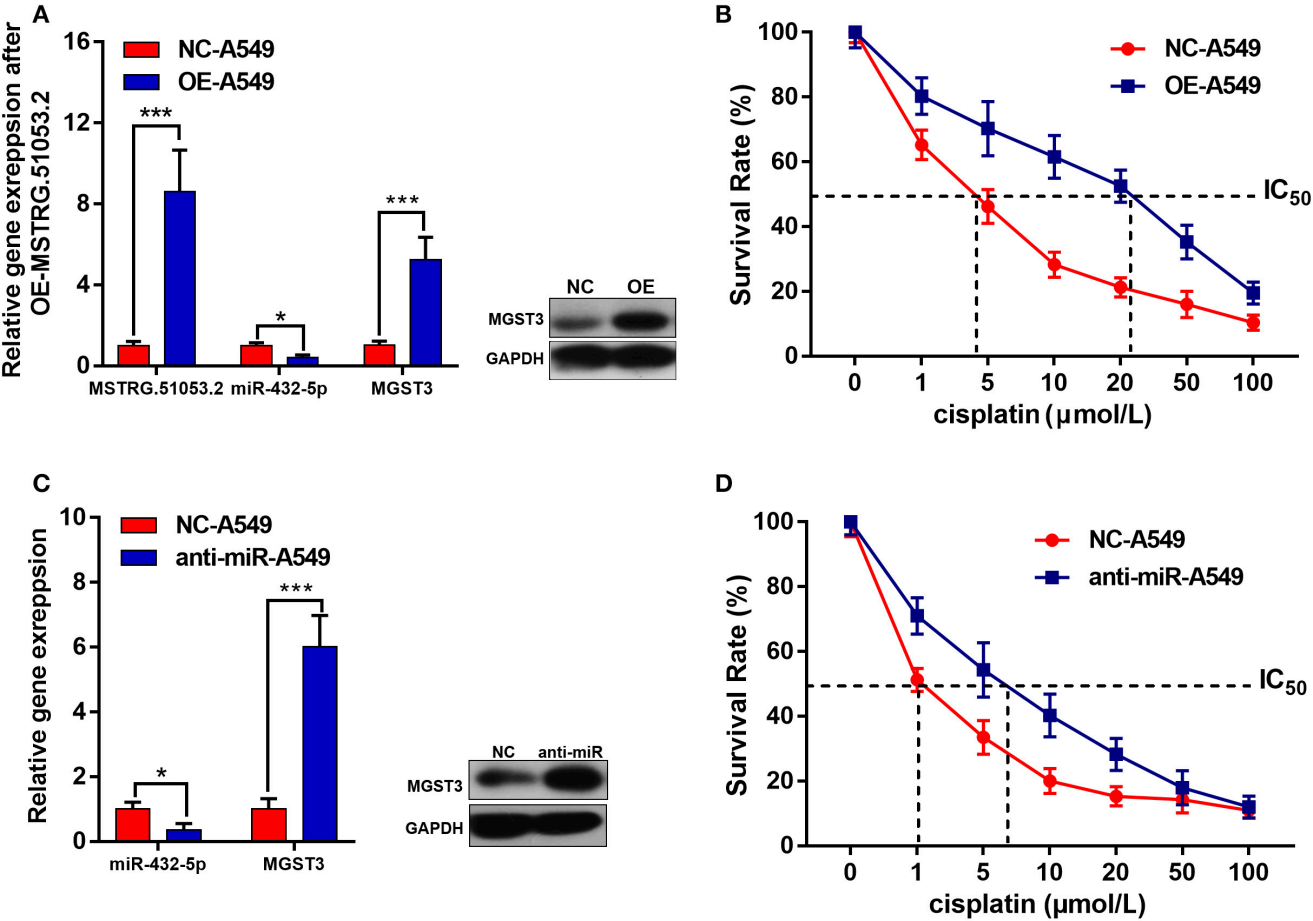
**(A)** The expression levels of MSTRG510532, miR-432-5p and *MGST3* after transfection with full-length MSTRG.51053.2 cDNA. **(B)** The IC_50_ value of cisplatin in the A549 cells overexpressing MSTRG.51053.2 were significantly higher than that in the control group. **(C)** The expression levels of miR-432-5p and *MGST3* after transfection with miR-432-5p inhibitor. **(D)** The IC_50_ value of cisplatin in the A549 cells treated with miR-432-5p inhibitor was significantly higher than that in the control group. *P < 0.05, ***P < 0.001.

As shown in Figure 6A, the expression level of MSTRG.51053.2 significantly increased after transfecting the cells with the full-length MSTRG.51053.2 cDNA. Additionally, the expression level of miR-432-5p significantly decreased, while that of MGST3 increased upon overexpression of MSTRG.51053.2. Moreover, the IC_50_ value of DDP in the A549 cells overexpressing MSTRG.51053.2 (25.32 ± 1.68 μmol/L) was significantly higher than that in the negative control (NC) group (4.26 ± 1.31 μmol/L) (Figure 6B).

### Effects of miR-432-5p Inhibition on the Expression of MGST3 and on DDP Sensitivity of A549 Cells

The expression level of miR-432-5p significantly decreased after transfecting the cells with the miR-432-5p inhibitor. The expression level of MGST3 significantly increased upon transfection with the miR-432-5p inhibitor (Figure 6C). Furthermore, the IC_50_ value of DDP in the miR-432-5p inhibitor-transfected A549 cells (6.98 ± 2.06 μmol/L) was significantly higher than that in the NC group (1.08 ± 0.55 μmol/L) (Figure 6D).

### Verification of ceRNA Regulatory Network of MSTRG.51053.2-miR-432-5p- MGST3

**Figure 7:**
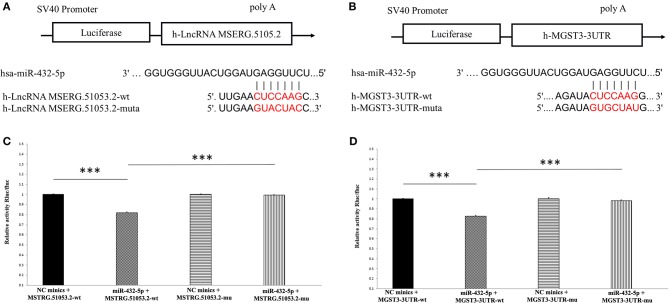
Representative fluorescent *in situ* hybridization (FISH) images in the blank group (blank) and the MSTRG.51053.2 and miR-432-5p co-transfection group (miR-432-5p mimic).

Fluorescent *in situ* hybridization (FISH) analysis was used to detect the co-localization of MSTRG.51053.2 and miR-432-5p in the A549 cells. The analysis revealed that MSTRG.51053.2 and miR-432-5p were mainly expressed in the cytoplasm and exhibited partial binding (Figure 7).

**Figure 8:**
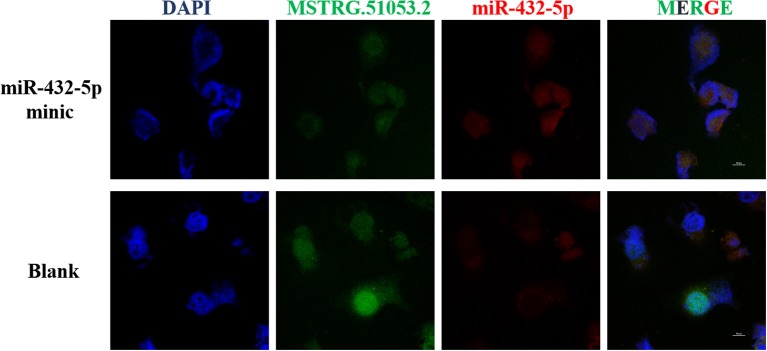
**(A,B)** The target sites of MSTRG51053.2 and MGST3 binding to miR-432-5p. **(C)** The luciferase activity of MSERG.5105.2-wild type was significantly inhibited in the miR-432-5p-transfected HEK293 cells. However, miR-432-5p transfection exhibited no inhibitory effect on the luciferase activity of MSERG.5105.2-mutant. **(D)** The luciferase activity of MGST3-wild type significantly decreased in the miR-432-5p-transfected HEK293 cells. However, miR-432-5p transfection exhibited no inhibitory effect on the luciferase activity of MGST3-mutant. ***P < 0.001.

The target sites of MSTRG.51053.2 and MGST3 binding to miR-432-5p are shown in Figures 8A,B. The luciferase activity of MSERG.5105.2-wild type was significantly inhibited in the miR-432-5p-transfected HEK293 cells. However, transfection with miR-432-5p exhibited no inhibitory effect on the luciferase activity of MSERG.5105.2-mutant type (Figure 8C). Similarly, the luciferase activity of MGST3-wild type significantly decreased in the miR-432-5p-transfected HEK293 cells, and transfection with miR-432-5p exhibited no inhibitory effect on the luciferase activity of MGST3-mutant type (Figure 8D).

## Discussion

In this study, we evaluated six pathways associated with conferring DDP resistance to tumor cells based on the differentially expressed genes and pathway enrichment analysis. The analysis revealed 17 related mRNAs, which were verified by qRT-PCR in the cells and tumor tissues. Eight differentially expressed mRNAs were further verified by western blotting. Western blotting analysis revealed that six proteins (MGST1, MGST3, ABCG2, FXYD2, ALDH3A1, and GST-ω1) were differentially expressed among G1-G4 DDP-resistant tumor tissues. Among the six genes encoding these proteins, ABCG2, ALDH3A1, MGST3, and FXYD2 were observed to exhibit interaction with the ceRNA regulatory network comprising 8 lncRNAs and 4 miRNAs. The expression levels of 8 lncRNAs and 4 miRNAs were quantified in the cells. The differential expression of 6 lncRNAs and 4 miRNAs was observed between A549/DDP and A549 groups, which was used to construct a ceRNA network. From the ceRNA network, we selected the MSTRG51053.2-miR-432-5p-MGST3 and PPAN-miR-665-ABCG2 regulatory networks for further analysis. Finally, the ceRNA regulatory network of MSTRG.51053.2-miR-432-5p-MGST3 was validated by luciferase reporter assay.

MGST1 (microsomal glutathione S-transferase 1), MGST3, and GST-ω1 (glutathione S-transferase ω1) are the members of glutathione S-transferases (GSTs). GSTs are crucial detoxification enzymes that catalyze the binding of substrates to glutathione during the second phase of detoxification, which can be activated by drug metabolites and sulfenic acid formation (16). DDP is readily detoxified by glutathione in the drug-resistant cells due to the overexpression of GSTs. Thus, GSTs are reported to be potential drug targets to reverse the DDP resistance in tumor cells (17). However, the roles of MGST1, MGST3 and GST-ω1 in DDP resistance have not been reported. In this study, the expression levels of MGST1, MGST3 and GST-ω1 were demonstrated to be upregulated in the A549/DDP group when compared to those in the A549 group. Additionally, these genes were upregulated among the G1-G4 DDP-resistant tumor tissues. These findings suggested that MGST1, MGST3 and GST-ω1 can serve as DDP drug targets to reverse the DDP resistance in NSCLC.

Moreover, MGST3 is involved in the ceRNA regulatory network of MSTRG.51053.2-miR-432-5p-MGST3. The role of MSTRG.51053.2 and miR-432-5p in conferring DDR resistance to cancer cells has not been previously investigated to the best of our knowledge. The miR-432-5p is reported to serve as a tumor suppressor in hepatocellular carcinoma and prostate cancer (18, 19). In this study, we not only detected the expression levels of the three genes in cells, tumor tissues of animals and patients, but also verified the regulatory relationship between MSTRG.51053.2 and miR-432-5p and that between MGST3 and miR-432-5p through luciferase reporter assay. Our study suggested that the lncRNA MSTRG.51053.2 may function as a ceRNA for miR-432-5p to regulate the DDP resistance in NSCLC.

ABCG2 (ATP binding cassette subfamily G member 2) is a member of ABC proteins family, a major drug transporter, which plays an important role in protecting the side population cells from cytotoxic drugs, like cisplatin. Additionally, ABCG2 is involved in multidrug resistance (20, 21). Although cisplatin is not an ABCG2 substrate, several studies have demonstrated that ABCG2 is a predictor of poor clinical prognosis of advanced NSCLC (22, 23). In this study, ABCG2 was demonstrated to be involved in the ceRNA regulatory network of PPAN-miR-665-ABCG2. A recent study reported that miR-665 could suppress the metastasis and invasion of osteosarcoma (24). Additionally, the serum exosomal miR-665 level in patients with hepatocellular carcinoma is markedly higher than that of the healthy control group, and is closely correlated with the clinical stage, tumor differentiation, and patient survival (25). However, the role of miR-665 in DDP resistance has not been reported. Our results demonstrated that miR-665 was significantly downregulated and PPAN was upregulated in the A549/DDP group when compared to those in the A549 group. These results concurred with those of animal experiments. The differential expression of PPAN, miR-665, and ABCG2 in the DDP-resistant cells and tissue suggested that this ceRNA regulatory network may be a potential mechanism underlying DDP resistance in NSCLC.

In conclusion, our study indicates that lncRNA MSTRG.51053.2 may function as a ceRNA for miR-432-5p to regulate the DDP resistance in NSCLC. Additionally, the MGST1, MGST3, GST-ω1, and ABCG2 mRNAs, miR-432-5p and miR-665 miRNAs, and MSTRG.51053.2 and PPAN lncRNAs can serve as DDP drug targets to reverse the DDP resistance in NSCLC.

## Materials and Methods

### Cell Culture and Treatment

Human lung adenocarcinoma cell line (A549) and DDP-resistant lung adenocarcinoma cell line (A549/DDP) were purchased from the Shanghai MeiXuan Biological Science and Technology Ltd. (Shanghai, China). The cell lines were cultured in RPMI-1640 medium supplemented with 10% fetal calf serum, streptomycin (100 mg/mL), and penicillin (100 μg/mL) at 5% CO_2_ and 37°C. The medium used for culturing A549/DDP cells was supplemented with 1 μg/mL DDP to maintain the drug-resistant phenotype. The cells in logarithmic phase were used for all experimental analyses.

### Library Preparation for lncRNA Sequencing

The lncRNA libraries were constructed using the rRNA-depleted RNA sample and NEBNext® Ultra™ Directional RNA Library Prep Kit for Illumina® (NEB, USA). The divalent cations were used for fragmentation. The synthesis of the first-strand cDNA was performed using M-MuLV reverse transcriptase and a random hexamer primer. The second-strand cDNA synthesis was performed using RNase H and DNA polymerase I. The 3′-end of the DNA fragments was adenylated and ligated with the NEBNext adaptor containing a hairpin-loop structure. AMPure XP system was used to select the cDNA fragments with a size range of 150–200 bp. The cDNA libraries were sequenced on an Illumina HiSeq 4000 platform.

### Library Preparation for Small RNA Sequencing

Small RNA library was prepared using the NEBNext® Multiplex Small RNA Library Prep Set for Illumina®. Briefly, the NEB 3′ SR adaptor was ligated to the 3′-end of miRNAs. The single-stranded DNA adaptor was converted into a double-stranded DNA molecule. The 5′-end adaptor was ligated to the 5′-end of miRNAs. The DNA fragments of size 140–160 bp were dissolved in 8 μL of an elution buffer. The libraries were sequenced on an Illumina HiSeq 2500 platform.

### Quality Control and Alignment

The raw reads were processed using Trimmomatic (v3.6) (26). Clean reads were selected after removing the low-quality reads and reads with length <35 bp. For miRNA-seq data, the reads with length <16 bp were removed.

### lncRNA-seq Data Processing

The clean reads were mapped to the Hg38 ENSEMBL (27) reference human genome using HISAT2 (28). The transcripts were reassembled using the Cufflinks tool (v2.2.1) (29). The gene expression levels of individual samples were quantified using the cuffquant (29) and cuffnorm (29) tools. The differentially expressed genes between A549/DDP and A549 groups were calculated using the cuffdiff (29) tool. The differentially expressed genes were considered statistically significant when |log fold change (FC)| > 1 and *q-*value < 0.01. Based on the genome annotation data, the genes were divided into lncRNAs and protein coding genes (mRNAs).

The differentially expressed mRNAs were subjected to KEGG pathway analysis using the clusterProfiler package (30). The enrichment was considered statistically significant when the *p*-value was < 0.05 after hypergeometric test.

### miRNA-seq Data Analysis

The clean reads were mapped to the human reference genome (ENSEMBL) using the Bowtie (v0.12.9) (13) software. Based on the known miRNA annotation information in the miRBase (14), the number of reads of the mature miRNA in each sample were obtained using the HTSeq (v0.9.1) (17) tool. The expression levels of miRNAs were quantified by count per million method. The prediction and quantification of new miRNAs were performed using the miRDeep2 software (v2.05). The differentially expressed miRNAs were selected using the quasi-likelihood F-test method in edgeR with the thresholds of |log FC| > 1 and false discovery rate <0.01.

### ceRNA Network Construction

The mRNAs involved in the pathways associated with conferring DDP resistance to tumor cells were selected for ceRNA network construction. The binding sites in 3′-UTR between differentially expressed miRNAs and the selected mRNA were predicted using miRanda (31). The miRNAs involved in the miRNA-mRNA pairs were selected to predict the miRNA-lncRNA interaction pairs using miRanda. The lncRNAs associated with the selected mRNAs were predicted by PC method (32) in the miRsponge (33). The PC method was based on two criteria: (1) the mRNA and lncRNA must have shared miRNA, and (2) the mRNA and lncRNA expression must conform to the positive co-expression. Finally, based on the miRNA-mRNA, mRNA-lncRNA, and miRNA-lncRNA pairs, the ceRNA network was constructed using Cytoscape (34).

### Xenograft of Nude Mice Resistant to DDP

**Figure 9:**
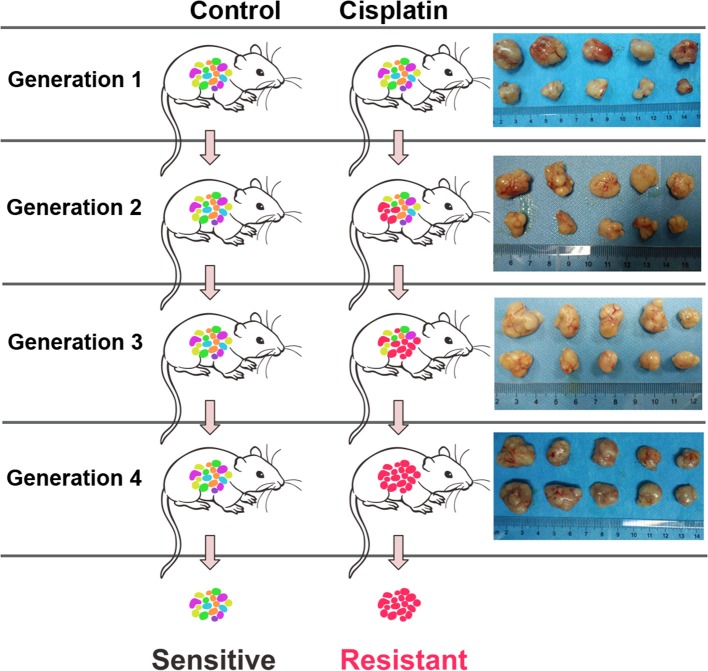
Generation of cisplatin-resistant nude mice strain by xenografting.

Male athymic nude mice (BALB/c-nu/nu) (5-week-old) were maintained in a specific pathogen-free condition under 12 h light/dark cycle and fed with standard chow diet and water. The logarithmic phase A549 cells (200 μL) at a cell density of 2 × 10^7^/mL were suspended in Matrigel and subcutaneously injected into the right flank of mice. The tumor diameter was measured at day 10 post-inoculation. The tumor volume was calculated using the following equation: V = ab^2^π/6 where, “a” represents maximum diameter, “b” represents minimum diameter, and “V” is the volume of tumor. The mice were divided into two groups, DDP group (intraperitoneal injection of cisplatin; 4 mg/kg body weight, once every 2 days) and control group (intraperitoneal injection of normal saline). The mice were humanely euthanized and their tumor tissues were collected. A sample of the tumor tissue was stored in liquid nitrogen, while some tissue samples were continuously passaged. After 4 generations, the xenografted DDP-resistant tumor tissue was obtained (Figure 9). All animal experiments were approved by the Institutional Animal Care and Use Committee of the Affiliated Huai'an Hospital of Xuzhou Medical University.

### Patients

We collected the lung tumor tissues from 17 patients with NSCLC who exhibited postoperative tumor recurrence at the Affiliated Huai'an Hospital of Xuzhou Medical University between January 2014 and October 2018. These patients underwent DDP-based chemotherapy after postoperative recurrence. The patients were classified as DDP-sensitive group (responder group, *n* = 10) and DDP-resistant group (non-responder group, *n* = 7) based on the sensitivity to chemotherapy.

### RNA Extraction

Total RNA was extracted from the cells using Trizol reagent. RNA purity was determined using the NanoPhotometer® spectrophotometer (Implen, CA, USA). RNA concentration was measured using the Qubit® RNA Assay Kit and Qubit® 2.0 Fluorometer (Life Technologies, CA, USA), while the RNA integrity was evaluated using the RNA Nano 6000 Assay Kit and Bioanalyzer 2100 system (Agilent Technologies, CA, USA).

### qRT-PCR Analysis

Total RNA was extracted from the cells, xenografted DDP-resistant tumors (G1-G4), and clinical tissue samples using Trizol reagent. The cDNA was synthesized using the TaKaRa reverse transcription kit (Takara, Dalian, China). The qRT-PCR analysis for mRNA and lncRNA was performed using the TaKaRa PCR detection kit (Takara) with GAPDH as an internal control. The qRT-PCR analysis for miRNA was performed using the miRNA detection kit (RiboBio, Guanghzou, China) with small nuclear RNA U6 as an internal control. The relative gene expression level was calculated using the 2^−ΔΔ*Ct*^ method.

### Western Blot Analysis

Protein was extracted from the xenografted DDP-resistant tumors (G1-G4) using RIPA lysis buffer. The extracted protein (20 μg) was subjected to sodium dodecyl sulfate-polyacrylamide gel electrophoresis using 12% gel. The proteins were transferred onto a polyvinylidene fluoride membrane. The membrane was blocked using 5% non-fat milk for 1 h. The membrane was probed with the primary antibodies (HMGCR, GST-ω1, GST-π1, ALDH3A1, MGST1, FXYD2, ABCG2, and MGST3; 1:1000) at 4°C overnight. Further, the membrane was probed with the horseradish-peroxidase labeled secondary antibodies (1:5000) at 37°C for 1 h. The proteins were visualized using enhanced chemiluminescence (ECL) reagents.

### Cell Transfection

The MSTRG.51053.2 (OE-MSTRG.51053.2) was overexpressed in the A549 cells by transfecting the full-length MSTRG.51053.2 cDNA (RiboBio) and its NC into the A549 cells. The miR-432-5p inhibitor (anti-miR-432-5p) and its NC were purchased from Sangon (Shanghai, China). The transfections were performed using Lipofectamine 2000 (Invitrogen, CA, USA).

### CCK-8 Assay

CCK-8 assay was used to investigate the effect of MSTRG.51053.2 overexpression and miR-432-5p inhibition on cell survival at different concentrations of DDP to calculate the IC_50_ value of DDP using the Cell Counting Kit-8 (CCK-8, Dojindo Molecular Technologies, MD, USA). Briefly, the cells were transferred to a 96-well plate at a cell density of 5 × 10^3^ cells/well. The cells were treated with different concentrations of DDP. Next, the cells were incubated with CCK-8 solution for 1 h at 37°C (5% CO_2_). The absorbance of the mixture was measured at 450 nm using a microplate reader (Bio-Rad, Hercules, CA, USA).

### Fluorescence *in situ* Hybridization (FISH) Assay

Co-localization of MSTRG.51053.2 and miR-432-5p in the A549 cells was detected using FISH assay. The cells were divided into two groups: the blank group (blank) and miR-432-5p minic transfection group (miR-432-5p minics). The probe for MSTRG.51053.2 was 5′-AAATCTCTTAAGTGATGGTAGGGTGATCTCAA-3′, while that for miR-432-5p was 5′-CCACCCAATGACCTACTCCAAGA-3′. Briefly, the cell suspension (5 × 10^4^ cells/well) was incubated on unstained slides in 24-well culture plates at 37°C and 5% CO_2_. The cells were washed with phosphate buffer saline (PBS) for 5 min and fixed in 4% paraformaldehyde. The cells were then washed thrice with PBS. The cells were incubated with 1 mL of pre-cooling transparent liquid for 5 min and washed with PBS. Next, the cells were blocked with 200 μL pre-hybridization solution for 30 min. Under dark conditions, 2.5 μL of 20 μM lncRNA FISH Probe Mix or internal reference FISH Probe Mix was added to 100 μL hybridization solution. The pre-hybridization solution in each well was discarded and 100 μL of probe hybridization solution containing the probes was added for hybridization at 37°C overnight in a dark moist chamber. After post-hybridization washes, the slides were stained with 4′,6-diamidino-2-phenylindole (DAPI) for 10 min. The slides were visualized under the Leica SP8 Multiphoton Confocal Microscope (Leica, Germany).

### Reporter Vector Construction and Luciferase Reporter Assay

The MSERG.5105.2 fragment that contains the predicted miR-432-5p binding site was amplified by PCR. The amplified fragment was cloned into a pmirGlO Dual-luciferase miRNA target expression vector to generate the reporter vector MSERG.5105.2-wild-type (MSERG.5105.2-wt). The putative binding site of miR-432-5p in MSERG.5105.2 was mutated by replacing the sequences of the putative binding site, which was named as MSERG.5105.2-mutated-type (MSERG.5105.2-mu). The vectors and miR-432-5p were co-transfected into the HEK 293T cells. The luciferase activity in the co-transfected cells was detected using the Dual-Luciferase Reporter Assay System (Promega). A similar method was used to determine the regulatory relationship between miR-432-5p and MGST3.

### Statistical Analysis

All statistical analyses were performed in SPSS 19.0 and Graphpad Prism 7.0. The data are expressed as mean ± standard deviation. Double-tailed *t*-test was used for the comparison between the two groups. One-way analysis of variance was used to compare the differences among three or more groups, while *q*-test was used for pairwise comparison between groups. The difference was considered statistically significant when the *P*-value was < 0.05.

## Data Availability Statement

The datasets generated for this study can be found in the NCBI Gene Expression Omnibus (GSE144520).

## Ethics Statement

The studies involving human participants were reviewed and approved by Animal Care and Use Committee of the Affiliated Huai'an Hospital of Xuzhou Medical University. The patients/participants provided their written informed consent to participate in this study. The animal study was reviewed and approved by Animal Care and Use Committee of the Affiliated Huai'an Hospital of Xuzhou Medical University.

## Author Contributions

YWan, YZhe, and WS conceived and designed the experiments. JZ, CX, YG, YWang, ZD, and YZha performed the experiments and analyzed the data. JZ and CX drafted the manuscript. All authors read and approved the final manuscript.

### Conflict of Interest

The authors declare that the research was conducted in the absence of any commercial or financial relationships that could be construed as a potential conflict of interest.
